# Does parental phubbing aggravates adolescent sleep quality problems?

**DOI:** 10.3389/fpsyg.2023.1094488

**Published:** 2023-02-06

**Authors:** Qian Ding, Siwei Dong, Yongxin Zhang

**Affiliations:** ^1^School of Education Science, Xinyang Normal University, Xinyang, China; ^2^China Key Laboratory of Adolescent Cyberpsychology and Behavior, Ministry of Education, Wuhan, China

**Keywords:** parental phubbing, negative emotions, sleep quality problems, self-control, adolescents

## Abstract

**Objective:**

Based on the theoretical model for the “stress–sleep” relationship, this study investigated the impact of parental phubbing on adolescent sleep quality problems and a moderated mediation mechanism.

**Methods:**

A total of 781 adolescents was surveyed using the Chinese version of Parental Phubbing Scale, the Ultra-brief Screening Scale for Depression and Anxiety Scale, the Self-Control Questionnaire for Chinese children, and the Chinese version of Pittsburgh Sleep Quality Index Scale.

**Results:**

Parental phubbing and negative emotions were significantly and positively correlated to sleep quality problems, but self-control was not correlated to sleep quality problems. Parental phubbing directly influenced sleep quality problems and also indirectly influenced sleep quality problems through the mediating effect of negative emotions. Moreover, self-control played a moderating role in the path of parental phubbing affecting negative emotions. That is, the effect was more significant for adolescents low in self-control relative to those high in self-control.

**Conclusion:**

Parental phubbing is a risk factor for adolescent sleep quality problems. This study is the first to demonstrate empirical evidence for the relationship between parental phubbing and sleep quality problems.

## 1. Introduction

With the development of information technology, cell phones are becoming more and more popular in modern society. Parental phubbing is an emerging issue in family context, which is defined as parents’ concentrating on using cell phones while neglecting their children in parent–child interaction (Xie and Xie, 2020; Zhang et al., 2021). Previous research has found that parental phubbing negatively affected adolescent mental health (Wang and Qiao, 2022; Xiao and Zheng, 2022), behavior (Wang et al., 2020; Zhang et al., 2021), and academic performance (He et al., 2022; Wang X. et al., 2022). Because of the salient negative effects of parental phubbing on adolescents, it is necessary to expand the research in this area. However, the effect of parental phubbing on physical health, which is equally important for adolescent health development, has not been studied. Good sleep quality is an important indicator of the physiological health of adolescents, and sleep quality problems could hamper adolescent health development (Wang et al., 2016). Sleep quality problems include insomnia, frequent nightmares, staying up late, and lack of sleep (Wang W. et al., 2022). A meta-analysis study showed that adolescents around the world had varying degrees of sleep quality problems (Gradisar et al., 2011). In China, children’s sleep quality is reported to be severely poor. According to a white paper released by the Chinese Sleep Research Society in March 2019, nearly 63% of Chinese children and adolescents aged between 6 and 17 years slept less than 8 h per day. And a recent meta-analysis has found that 20% adolescents in junior high school and 28% adolescents in senior high school have sleep disturbances (Liang et al., 2021). Therefore, the present study would examine the effect of parental phubbing on adolescent sleep quality problems.

### 1.1. Parental phubbing and adolescent sleep quality problems

Although there is no direct evidence to support the idea that parental phubbing increases adolescent sleep quality problems, this assumption can be extrapolated from empirical results in previous research and the theoretical model of the “stress – sleep” relationship. The theoretical model of the “stress – sleep” relationship contends that stressors (family, social, and work) have a great impact on individuals’ sleep quality, and stress response and stress coping are important in regulating the relationship between stress and sleep quality (Yan et al., 2010). According to the theoretical model of the “stress – sleep” relationship and many empirical studies (Liu et al., 2017; Yeung et al., 2017; Wang W. et al., 2022), stress is one of the most common precipitants of sleep quality problems. As parental phubbing has been prevalent in recent years (Hong et al., 2019; Zhang et al., 2021) and 35% parents in a study have reported frequently using their mobile phones while interacting with their children (Kildare and Middlemiss, 2017), parental phubbing may have become one of adolescent main stressors today. If parents frequently use mobile phones, the ongoing parent–child communication would be interrupted, resulting in parents not being able to respond to their children’s needs in time. Specifically, in parent–child interaction, parental phubbing prevents adolescents from normally enjoying parental attention and companionship (Xiao and Zheng, 2022), and it directly decreases the quality of parent–child relationships and family cohesion (Niu et al., 2020; He et al., 2022). Moreover, some researchers pointed out that phubbing is a kind of social exclusion behavior (David and Roberts, 2017; Xie and Xie, 2020), and chronic experience of social exclusion could be regarded as a stressful situation (Wang H. et al., 2017). Thus, parental phubbing as a kind of stressor may lead to sleep quality problems in adolescents. Therefore, this study proposes hypothesis 1 (H1): Parental phubbing has a significant positive effect on adolescent sleep quality problems.

In addition, the mechanism by which stress affects sleep quality may be influenced by other factors. According to the theoretical model of the “stress – sleep” relationship, stressors can also affect sleep quality through the emotional responses and stress coping of individuals (Yan et al., 2010). Therefore, to further discuss the influence of parental phubbing on adolescent sleep quality problems, this study will test emotional responses as a mediator and stress coping as a moderator on the relationship between parental phubbing and adolescent sleep quality problems grounded on this theoretical model.

### 1.2. Negative emotions as the mediator

According to the theoretical model of the “stress – sleep” relationship, stressors are not only correlated to sleep quality, but also indirectly correlated to sleep quality through the mediation of stress responses such as physiological responses, cognitive responses, and emotional responses (Yan et al., 2010). Considering that negative emotions, for example, depression and anxiety, are common results of parental phubbing (Xie and Xie, 2020; Wang et al., 2021; Wei et al., 2022; Xiao and Zheng, 2022), we speculate that negative emotions as typical emotional responses mediate the relationship between parental phubbing and adolescent sleep quality problems.

On the one hand, children’s healthy development of social-emotions relies on the reality that parents are responsive and sensitive to children’s needs during parent–child interactions (Swain et al., 2010). However, parental phubbing renders it difficult for parents to identify and respond to their children’s various needs in time, leading to a series of emotional problems for their children. For example, some studies indicated that adolescents expect their parents to be highly involved in parent–child interactions (He et al., 2022; Xiao and Zheng, 2022). In this case, adolescents may develop negative emotions if treated by parental phubbing, a form of social exclusion and rejection usually unaware by parents (David and Roberts, 2017; Xie and Xie, 2020). In addition, a series of previous studies have confirmed that parental phubbing is positively associated with negative emotions such as depression and anxiety in adolescents (Wang et al., 2021; Xiao and Zheng, 2022).

On the other hand, based on the theoretical model of the “stress – sleep” relationship, the emotional response is one of the ways by which stress acts on sleep quality (Yan et al., 2010). This argument is supported by some empirical studies. For example, a recent study has shown that stressful life events increases negative emotions thereby indirectly influencing sleep quality (Wang and Matsuda, 2021). Compared to stressful life events, depressive emotional response was a stronger predictor for sleep disturbance among women with metastatic breast cancer (Palesh et al., 2007). Furthermore, numerous studies have agreed on the result that negative emotions (e.g., anxiety, depression) are important triggers of sleep quality problems (Guo and Sun, 2016; Bjorøy et al., 2020; Wang and Matsuda, 2021; Wang W. et al., 2022). Therefore, the present study proposes hypothesis 2 (H2): Negative emotions mediate the association between parental phubbing and sleep quality problems.

### 1.3. Self-control as the moderator

Additionally, the theoretical model of the “stress – sleep” relationship suggested stress coping (e.g., cognitive regulation, emotion regulation, coping style, social support and personalities) may moderate the relationship between stressful events (e.g., parental phubbing) and stress response, sleep quality (Yan et al., 2010). Likewise, the stress and coping theory contends that cognitive and emotional control can mitigate or even eliminate the stressors’ effects (Lazarus and Folkman, 1984). Both theories prompt that individuals’ capacity for cognitive and emotional regulation/control may moderate the effects of parental phubbing on negative emotions. Self-control, which refers to the ability to modify and regulate their behavior, thoughts, and emotions to conform to social or ego standards (Muraven and Baumeister, 2000; Tangney et al., 2004), was found to be an important factor that buffers the negative influence of stressful events on individuals’ stress response (Rosenbaum, 1989; Niu et al., 2020; Schnell and Krampe, 2021). When external stressors elicit stress responses in individuals, self-control can interrupt these responses and return the body to the normal state (Baumeister et al., 1998; Muraven et al., 1998). Therefore, we speculate that self-control may moderate the association between parental phubbing and adolescent negative emotions.

First, from the perspective of self-control resources, self-control represents a relatively stable resources in the individual resource pool (Hagger et al., 2010). Despite that social stress in the form of parental phubbing might cause adolescent negative emotions, adolescents higher in self-control have a higher baseline of total self-control resources, which means they are more capable to regulate their emotions and effectively control negative emotions at a lower level. Second, from the perspective of response patterns, individuals with different levels of self-control should respond differently to social stress (e.g., parental phubbing). As those adolescents higher in self-control have a higher ability to shift their attention from parental phubbing to other places (de Ridder et al., 2012), their negative emotions might maintain at a relatively lower level. Third, empirical studies have found that self-control moderated the relationship between COVID-19 stress and general mental distress such as depression and anxiety (Schnell and Krampe, 2021). Individuals high in self-control experience less emotional distress compared to those low in self-control (Gramzow et al., 2000). Accordingly, based on the above theories and empirical evidence, the present study proposes hypothesis 3 (H3): Self-control moderates the effect of parental phubbing on negative emotions.

### 1.4. The present study

**Figure 1:**
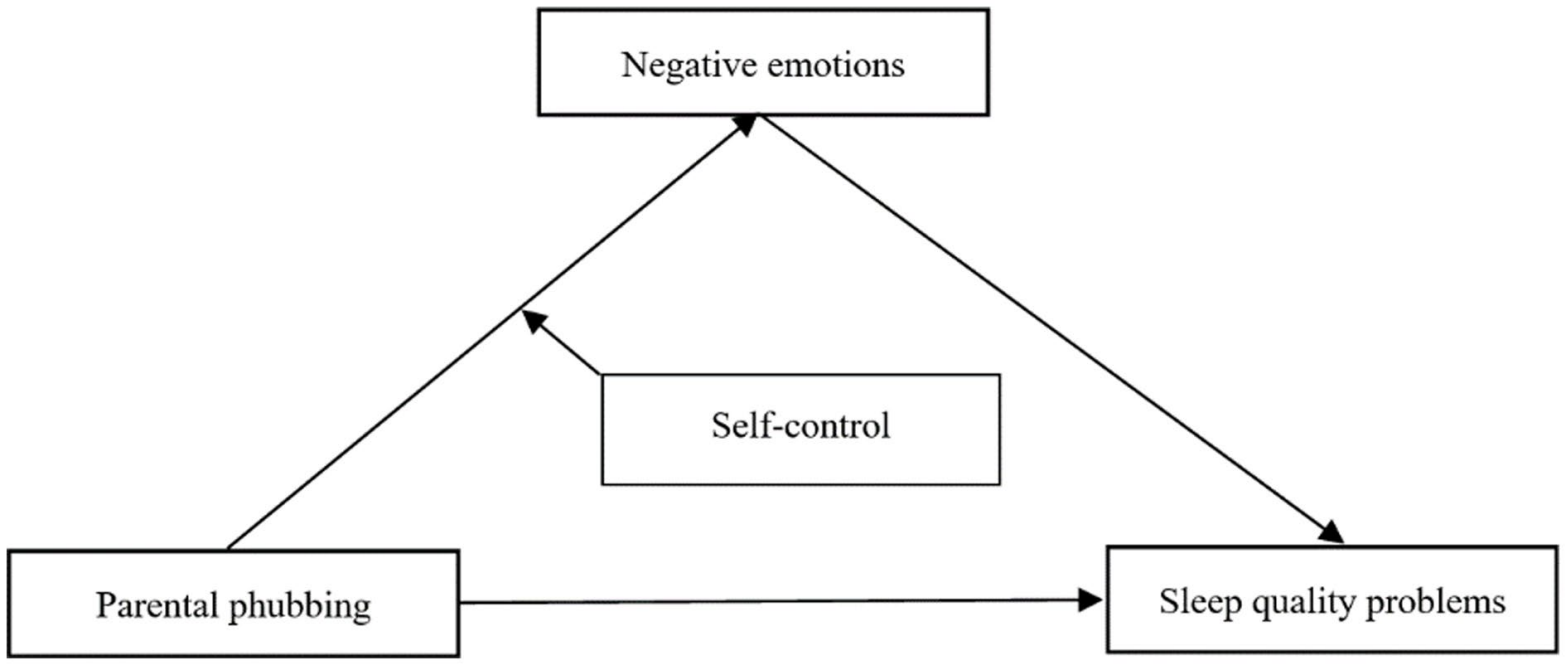
The hypothetical model.

In summary, based on the theoretical model of the “stress – sleep” relationship, the present study intends to investigate the relationship between parental phubbing, negative emotions, self-control and adolescent sleep quality problems and their mechanisms. We constructed a moderated mediating hypothesis model that parental phubbing significantly and positively predicted adolescent sleep quality problems through the mediation of negative emotions, and self-control moderated the first path of this mediating effect (Figure 1).

## 2. Methods

### 2.1. Participants and procedure

All the participants (Grades 7–12) came from two junior high schools and two senior high schools in central China through convenient sampling. Before the questionnaire investigation, we received permission to conduct this survey from the Ethical Committee for Scientific Research in our institution, and we sought consent from the school, teachers, and parents. The assessment was conducted in school classrooms. In the course of data collection, the trained graduate students gave standardized instructions in front of the class, and all of the participants were guaranteed that their answers would be kept confidential and used solely for academic research. Of 896 participants available on the day of the survey, 823 adolescents (all of them were living with their father, mother, or both in the last year, and their parents had at least one smartphone and regularly used it in the last year) participated in the study survey. After removing the invalid questionnaires with missing answers and consistent responses, 781 valid questionnaires were obtained, with an effective rate of 94.5%. Among the valid participants, 506 students were from rural areas and 275 students were from urban areas; 415 students were male and 366 students were female; 389 students were junior high school students and 392 students were senior high school students. Their ages ranged from 12 to 18, the average age was 14.89, and the standard deviation was 1.79. Parents’ education levels were very close to those in the latest census data in central China; 30.9% of the fathers and 25.0% of the mothers had a high school education or higher.

### 2.2. Measures

#### 2.2.1. Parental phubbing

Adopted the Parental Phubbing Scale revised by Ding et al. (2020). As a single dimension, the scale consists of 9 items (Roberts and David, 2016), such as “During a typical mealtime that my parents and I spend together, my parents pull out and check their cell phone.” Participants rated each item on a 5-point Likert scale from 1 (never) to 5 (always). Higher scores indicated more severe parental phubbing in parent–child interactions. Previous studies showed that the scale had good reliability and validity when used with Chinese adolescents (Zhang et al., 2021; He et al., 2022; Xiao and Zheng, 2022). The index of CFA based on the original structure showed a good fit: *χ*^2^/df = 4.63, CFI = 0.95, GFI = 0.96, TLI =0.94, RMSEA = 0.07. In this study, the Cronbach’s *α* value of the scale was 0.82.

#### 2.2.2. Negative emotions

Negative emotions were measured using the Ultra-brief Screening Scale for Depression and Anxiety Scale revised by Qian et al. (2021), which was adopted with the original Patient Health Questionnaire-4 (PHQ-4; Kroenke et al., 2009). The scale comprises 4 items, such as “Feeling nervous, anxious or on edge.” Participants rated each item from 0 (never) to 3 (almost daily). Higher scores indicated higher levels of negative emotions of depression and anxiety. The index of CFA based on the original structure showed a good fit: *χ*^2^/df = 3.98, CFI = 0.96, GFI = 0.95, TLI =0.98, RMSEA = 0.06. In this study, the Cronbach’s *α* value of the scale was 0.86.

#### 2.2.3. Self-control

The level of self-control was assessed using the Self-Control Questionnaire for Chinese children designed by Dong and Lin (2011). This questionnaire consists of 5 items. Each item is scored according to 1–4 points. With higher total scores indicating a higher level of self-control for the individual. Previous study had demonstrated the questionnaire’s good reliability and validity for Chinese adolescents (e.g., Zhu et al., 2018). The index of CFA based on the original structure showed a good fit: *χ*^2^/df = 5.47, CFI = 0.95, GFI = 0.98, TLI =0.89, RMSEA = 0.07. In this study, the Cronbach’s *α* value of this questionnaire was 0.76.

#### 2.2.4. Sleep quality problems

Sleep quality problems were measured using the Chinese version of Pittsburgh Sleep Quality Index scale (PSQI; Liu et al., 1996), which has good validity, internal consistency and test–retest reliability, and been widely used among Chinese adolescents (e.g., Deng et al., 2021; Wang W. et al., 2022; Ye et al., 2022). The PSQI comprises 18 self-rated items, including seven factors: subjective sleep quality, time to sleep, sleep duration, sleep efficiency, sleep disturbance, hypnotic medication, and daytime function. Each item is scored according to 0–3 points. The average scores on the PSQI ranged from 0 to 3, with higher scores indicating more sleep quality problems. The index of CFA based on the original structure showed a good fit: *χ*^2^/df = 5.44, CFI = 0.90, GFI = 0.97, TLI =0.88, RMSEA = 0.07. In this study, the Cronbach’s *α* value of the scale was 0.76.

### 2.3. Statistical analyzes

In this study, trained postgraduates majoring in psychology conducted the test on a class basis, and the questionnaires were distributed and collected on the spot. We used PROCESS version 3.0 (Hayes, 2013) to test the mediation and moderation models and performed descriptive statistics and correlation analyzes on SPSS 21.0. Significance testing of regression coefficients was performed using Bootstrap (sampling repeated 5,000 times) to obtain robust standard errors and a 95% bias-corrected confidence interval (CI) for parameter estimation. In addition, age and gender were included as control variables. The Harman single-factor test method was applied to process all measurement items through nonrotating exploratory factor analysis. According to the analytical results, there are a total of 7 common factors with eigenvalues greater than 1 extracted, and the first common factor can be used to explain 21.10% of the total change, which falls short of the 40% standard threshold. That is, there is no deviation caused by the same method for data collection in this study (Podsakoff et al., 2003).

## 3. Results

### 3.1. Preliminary analyzes

Pearson correlations as well as the means and standard deviations of the main variables were presented in Table 1. Parental phubbing was significantly and positively associated with negative emotions and sleep quality problems, while not associated with self-control. Negative emotions were significantly negatively associated with self-control and significantly positively associated with adolescent sleep quality problems. Self-control is not associated with sleep quality problems.

**Table 1:** Means, standard deviations and correlations for the main variables (*N* = 781).

Variables	*M*	*SD*	1	2	3	4
1. Parental phubbing	2.48	0.80	1			
2. Negative emotions	1.19	0.81	0.28^***^	1		
3. Self-control	2.64	0.60	−0.02	−0.08^*^	1	
4. Sleep quality problems	1.10	0.43	0.22^***^	0.54^***^	−0.06	1

*p < 0.05, ***p < 0.001.

### 3.2. Mediation and moderation analyzes

We used Model 4 of PROCESS (Hayes, 2013) to examine the possible association between parental phubbing and sleep quality problems as well as the possible mediating effect of negative emotions. After controlling for age and gender, we first found that parental phubbing positively predicted sleep quality problems, *β* = 0.20, *p* < 0.001. Second, parental phubbing positively predicted negative emotions, *β* = 0.27, *p* < 0.001; negative emotions positively predicted sleep quality problems, *β* = 0.49, *p* < 0.001. Third, the bias-corrected bootstrapping mediation test indicated that the process by which parental phubbing predicted sleep quality problems through negative emotions was significant, with indirect effect = 0.13, *SE* = 0.02, 95% CI = [0.09, 0.17]. The indirect effect (parental phubbing → negative emotions → sleep quality problems) accounted for 64.76% of the total effect. The results of the mediation analysis support H1 and H2.

**Figure 2:**
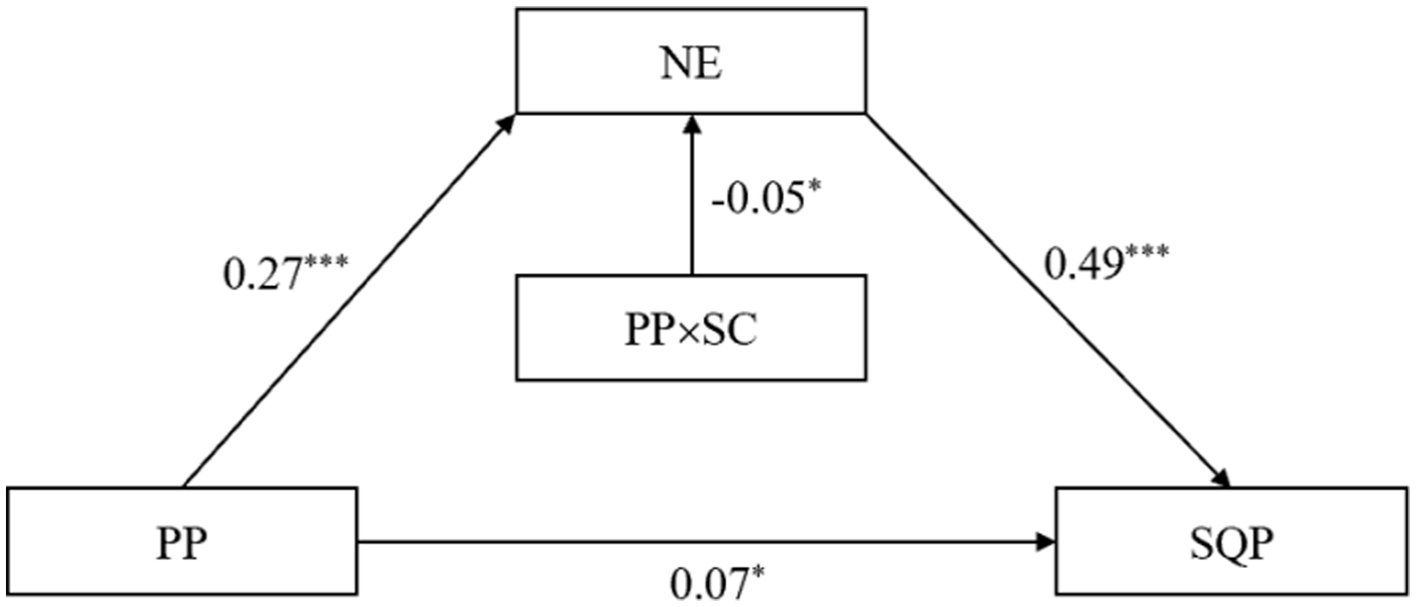
The integrated model. *p < 0.05, ***p < 0.001. PP, parental phubbing; NE, negative emotions; SQP, sleep quality problems; SC, Self-control.

We employed Model 7 of PROCESS (Hayes, 2013) to investigate whether self-control moderated the association between parental phubbing and negative emotions. Regression analysis indicated that parental phubbing positively predicted negative emotions (*β* = 0.27, *p* < 0.001) and self-control was not a significant predictor of negative emotions (*β* = −0.06, *p* > 0.05), while the interaction term between parental phubbing and self-control was a significant predictor of negative emotions [*β* = −0.05, *p* < 0.05, 95% CI (−0.10, −0.01)]. This result suggests that self-control moderates the first half of the mediating pathway“parental phubbing → negative emotions → sleep quality problems,” which supports H3. The results of the mediation and moderation analysis are presented in Table 2 and Figure 2.

**Table 2:** The mediation and moderation model.

Predictor	Criterion: NE				Criterion: SQP			
	*β*	*SE*	*t*	[LLCI, ULCI]	*β*	*SE*	*t*	[LLCI, ULCI]
Gender	−0.05	0.07	0.48	[−0.18, 0.09]	−0.02	0.06	−0.30	[−0.13, 0.10]
Age	0.09	0.02	4.54^***^	[0.05, 0.12]	0.09	0.02	5.64^***^	[0.06, 0.13]
PP	0.27	0.03	8.00^***^	[0.21, 0.34]	0.07	0.03	2.31^*^	[0.01, 0.13]
NE					0.49	0.03	15.64^***^	[0.43, 0.55]
SC	−0.06	0.04	0.09	[−0.13, 0.01]				
PP × SC	−0.05	0.03	−2.07^*^	[−0.10, −0.01]				
*R^2^*	0.11				0.32			
*F*	19.37^***^				91.46^***^			

*p < 0.05, ***p < 0.001; Gender: female = 0, male = 1; PP = parental phubbing; NE = negative emotions; SQP = sleep quality problems; SC = Self-control.

**Figure 3:**
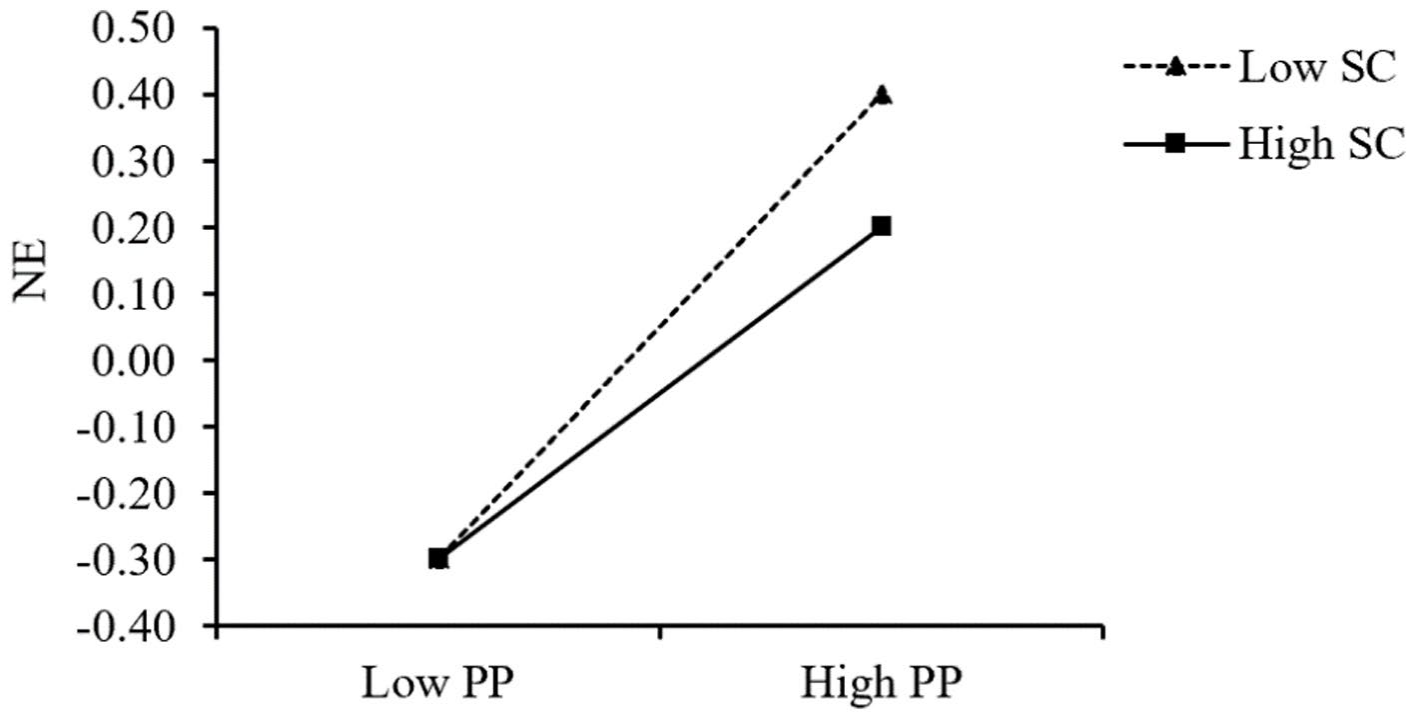
The interaction of parental phubbing and self-control on negative emotions. PP, parental phubbing; NE, negative emotions; SC, Self-control.

To more clearly reveal how self-control moderates the relationship between parental phubbing and negative emotions, self-control was divided into high and low groups by mean plus or minus one standard deviation, a simple slope test was conducted and an interaction plot was drawn (Figure 3). The predictive effect of parental phubbing on negative emotions was significant for adolescents low in self-control (*β*_simple_ = 0.33, *t* = 7.38, *p* < 0.001) and diminished for adolescents high in self-control (*β*_simple_ = 0.22, *t* = 5.36, *p* < 0.001).

Moreover, the results of PROCESS model 7 also showed the mediation mechanism was moderated by self-control [the index of moderated mediation = −0.03, Boot *SE* = 0.01, 95% CI = (−0.06, −0.01)], which indicated the significant differences in mediation effect sizes between the high self-control groups and low self-control groups. Specifically, for the low self-control adolescent group, the mediation effect of negative affect was 0.15 [Boot *SE* = 0.02, 95% CI = (0.11, 0.20)]; for the high self-control group, the mediation effect of negative affect was 0.10 [Boot *SE* = 0.02, 95% CI = (0.06, 0.15)].

## 4. Discussion

Parents usually neglect their children in social settings by concentrating on phone use and are unaware of the potential negative outcomes. In fact, a large number of correlational studies have revealed a positive association between parental phubbing and problem behaviors (Xie and Xie, 2020; Wang et al., 2021; Zhang et al., 2021; He et al., 2022; Wang and Qiao, 2022; Wei et al., 2022; Xiao and Zheng, 2022). Among these problems, mental health has received considerable attention from both the public and academics (Xie and Xie, 2020; Xiao and Zheng, 2022). However, no research to date has directly focused on the association between parental phubbing and adolescent physical health, such as sleep quality which has become an increasing burden on Chinese society now. To fill this gap, the current study examined the association as well as the mediator–negative emotions–to explain the emotional response mechanism underlying this association. Furthermore, we examined whether a protective factor for this mediating effect exists by introducing self-control as a moderator. The implications and limitations of the current study are discussed below.

### 4.1. Parental phubbing on adolescent sleep quality problems

First, the present study examined the relationship and potential mechanisms between parental phubbing and adolescent sleep quality problems. To our knowledge, the present study was the first to find a positive predictive effect of parental phubbing on adolescent sleep quality problems, validating research hypothesis H1. This result supports the theoretical model of the “stress – sleep” relationship (Yan et al., 2010) and the argument that stress is one of the most common factors of sleep quality problems (Liu et al., 2017; Yeung et al., 2017; Wang and Matsuda, 2021; Wang W. et al., 2022). For adolescent sleep quality, previous researches paid more attention to stressful life events (Brand et al., 2011; Wang and Matsuda, 2021), especially academic stress (Deng et al., 2021). Recently, some studies considered the COVID-19 pandemic and the policies to contain it as a stressor that adversely affected sleep quality (e.g., Wang W. et al., 2022). These factors are important and relatively obvious stressors for adolescent sleep quality, however, parental phubbing can easily be ignored. Based on relevant theoretical and empirical research (David and Roberts, 2017; Wang R. et al., 2017; Xie and Xie, 2020), this study regarded parental phubbing as a kind of social stress between parents and children, and found that parental phubbing significantly and positively predicted adolescent sleep quality problems, which contributes to the field of adolescent sleep quality problems by enriching the predictors. Unlike other negative parenting behaviors, parental phubbing as a technology-era product is characterized by covert nature. Previous negative parenting behaviors including violence and reprimand, are overt negative approaches and have been alerted to and avoided by the public. In contrast, parental phubbing is not accompanied by overt violence or conflict. Moreover, in the technology era, cell phone use is often justified by the need to work and relax. As a result, it is difficult for parents to realize their own mistakes when they engage in phubbing at home.

In addition, the present study further illustrated the harmful effects of parental phubbing, not only deepening the understanding of parental phubbing but also expanding the research on the effects of the technological era on adolescents’ physical and mental development. Previous studies (Xie and Xie, 2020; Wang et al., 2021; Zhang et al., 2021; He et al., 2022; Wang and Qiao, 2022; Wei et al., 2022; Xiao and Zheng, 2022) emphasized that parental phubbing, as a novel negative parent–children interaction, leads to adolescent internalizing and externalizing problems (e.g., anxiety, depression, cell phone addiction, cyberbullying). Our findings indicated that parental phubbing also had the potential to affect adolescent physiological health, resulting in sleep quality problems. Adolescent health development involves combined development of psychological and physical conditions. Moreover, having good quality of sleep helps to confront intense academic tasks. Therefore, along with previous studies on the negative effects of parental phubbing, we believe it is necessary and meaningful to draw attention to the negative effects of parental phubbing on adolescent sleep quality.

### 4.2. The mediation of negative emotions

Second, this study revealed that negative emotions are a mediator that explains the effect of parental phubbing on adolescent sleep quality problems, which proved research hypothesis H2. We adopted the theoretical model of “stress – sleep” relationship to explain the effect of parental phubbing on adolescent sleep quality problems. The theoretical model of “stress – sleep” relationship emphasizes the importance of stress response (e.g., emotional response) as a key risk factor in individuals’ sleep quality problems (Yan et al., 2010). In accordance with this theoretical model, our findings indicated that adolescents who are highly phubbed by their parents had experienced more anxiety and depression and therefore caused a higher likelihood of their sleep quality problems. To further test the universality of the results of this study, future research should examine whether other forms of stress responses (e.g., cognitive responses, behavioral responses) mediate the association between parental phubbing and sleep quality problems. For example, previous researches showed that a typical behavioral response to parental phubbing was adolescent problematic mobile phone use (Niu et al., 2020; Zhang et al., 2021), which had be found as a significant cause of sleep quality problems (Zou et al., 2019). It suggested that problematic mobile phone use as a kind of behavioral response might mediate the association between parental phubbing and adolescent sleep quality problems.

In accordance with the result of this study, previous studies have also found that negative emotions can play a mediating role in the relationship between stressors and sleep quality. For examples, a study by Zhao et al. (2021) found that anxiety mediated the relationship between perceived stress and sleep quality among the non-diseased general public in China during the COVID-19 pandemic. A study by Wang and Matsuda (2021) found that negative emotions play a mediating role in stressful life events and sleep quality among Chinese and Japanese undergraduate students. These results suggest that previous studies mainly focused on negative emotions mediating the associations between stressors related to overt life events and sleep quality. However, our research examined the mediating role of negative emotions by focusing on stressors related to parent–children interaction which is covert social stress. The results of this study expand our understanding of the mechanism of stressors affecting sleep quality. Additionally, it is worthy of noting that previous studies found sleep quality also affects adolescent emotional control functions, and adolescents with sleep quality problems showed more negative emotions such as anxiety and depression (Soffer-Dudek et al., 2011; Liu et al., 2022; Wang W. et al., 2022). In other words, negative emotions trigger sleep quality problems, and sleep quality problems in turn exacerbate negative emotions. Thus, if adolescents suffer from parental phubbing, they might also be caught in a vicious cycle of negative emotions and sleep quality problems, future research could use longitudinal design to explore this problem.

### 4.3. The moderation of self-control

Third, the present study also found that self-control moderated the relationship between parental phubbing and negative emotions, which supported the research hypothesis H3. The theoretical model of the “stress – sleep” relationship suggested stress coping, especially cognitive regulation and emotion regulation, may moderate the relationship between stressors and stress response (Yan et al., 2010). In line with previous studies that adopted self-control as a moderator (Niu et al., 2020; Schnell and Krampe, 2021), the present study also showed a protective effect of self-control in cases of stressors. Specifically, adolescents high in self-control had less negative emotions as a result of parental phubbing than those low in self-control. The stress and coping theory state that coping is an action taken to minimize perceived “threat” (Lazarus and Folkman, 1984). In terms of coping consequences, stress coping can be divided into positive coping and negative coping, positive coping is more mature and usually involves problem solving, help seeking, cognitive and emotional regulation; negative coping is immature and includes self-blame, fantasy, and avoidance (Yan et al., 2010).

Self-control, as a self-regulation ability, allows individuals to effectively control their thoughts, impulses, behaviors, and emotions to ensure that they minimize the impact of the “threat” (Tangney et al., 2004; Finkenauer et al., 2005). Thus, adolescents high in self-control are more capable of taking a positive coping to parental phubbing, such as controlling their thoughts of interpreting parental phubbing as social exclusion or parental rejection, controlling their negative emotions, and not overreacting. In addition, studies have shown that lower self-control is associated with lower psychological resilience. Psychological resilience is the ability of an individual to respond positively and return to a good state despite experiencing setbacks or adversity (Ge et al., 2021). Therefore, relative to adolescents higher in self-control, those lower in self-control have more difficulty controlling and regulating their emotions when experiencing parental phubbing, and they eventually fall into negative emotions and have difficulty recovering to a normal state, which leads to more sleep quality problems.

## 5. Theoretical and practical implications

The study has both theoretical and practical implications. To begin, this study has two theoretical implications. First, the present study was one of the studies that adopted both stress response and stress coping as mechanism in the influence of stress on sleep quality problems (Yan et al., 2010; Liu et al., 2020). Previous studies had demonstrated the effect of stress on adolescent sleep quality (Liu et al., 2017; Yeung et al., 2017), the present study identified a new stressor in the digital age, and further revealed how parental phubbing as a stressor affect sleep quality in adolescents through the mediation of emotional response (e.g., negative emotions) and the moderation of positive coping (e.g., self-control). The results contributed to the understanding of the detailed process of stress on sleep quality and supported the theoretical model of the “stress – sleep” relationship (Yan et al., 2010). Second, the present study expanded our understanding of the diversity of parental phubbing on adolescent physical and mental health. As we know, the present study was the first to discuss the relationship between parental phubbing and sleep quality problems. Previous research had shown that parental phubbing is positively associated with adolescent behavioral problems such as problematic smartphone use and cyberbullying perpetration (e.g., Wang et al., 2020; Zhang et al., 2021; Wei et al., 2022; Wang X. et al., 2022), psychological problems such as depression (e.g., Xie and Xie, 2020; Xiao and Zheng, 2022), and academic problems such as learning burnout (He et al., 2022; Wang X. et al., 2022). These results suggest that previous studies mainly focused on parental phubbing and adolescent mental health, however, the present study found that parental phubbing is also positively associated with adolescent sleep quality problems, which is a typical physical health topic.

In addition, this study has three practical implications. First, the results of this study suggested that parental phubbing, as a social stressor between parents and children, was a risk factor for adolescent sleep quality in recent days. Although cell phones have become an essential daily tool and phubbing has become a common occurrence, parents still need to appropriately manage the time and occasions of using cell phone at home. Second, the results of this study showed that proximal factors (e.g., negative emotions) have a more direct impact on sleep quality than distal context factors (e.g., parental phubbing). Thus, intervention strategies for adolescent emotional regulation may be more effective in improving sleep quality. Apart from reducing phubbing, parents also need to actively express care for and give adequate attention to their adolescent children and provide some encouragement and guidance to reduce adolescent negative emotions. Third, this study also found that self-control alleviated the influence of parental phubbing on adolescent negative emotions. Therefore, self-control should be given more attention for the intervention of adolescent negative emotions. Previous studies suggested that exerting self-control may consume self-control strength (Muraven and Baumeister, 2000). To maintain self-control, it is better to reduce other stressors in adolescent daily life. Furthermore, previous evidence showed that priming self-awareness and self-affirmation contributed to improving self-control (Schmeichel and Vohs, 2009; Alberts et al., 2011).

## 6. Limitations and future orientation

The present study also has several limitations. First, this study utilized a cross-sectional design, but individuals’ sleep quality fluctuated from day to day. Therefore, a diary and experience sampling research design which is characterized by collecting individuals’ immediate responses at multiple time points, could be considered for future studies to more accurately understand the effects of parental phubbing on adolescent sleep quality. Second, all variables in this study were measured by adolescent self-reports. Considering that adolescents are sensitive to self-esteem and emotions, they may conceal or exaggerate the true situation of emotion and sleep quality problems, future studies should adopt multiple measurements (e.g., reports from important others, such as parents, teachers, and friends; or other objective indicators, such as automatic recording through apps) to make it more comprehensive and objective. Third, the relationship between stress, emotions, and sleep quality is not unique. For example, some studies have found that sleep quality can also influence individuals’ stress perception and emotional experience (Liu et al., 2022; Wang W. et al., 2022). Therefore, future research can consider this perspective and examine multiple mechanisms between the three. Fourth, the subjects of this study are only Chinese adolescents, limiting its generalizability. As a collectivist society, Chinese culture emphasizes family relationships and social stress, it must be cautious about generalizing the findings of this study to other cultures (Wang et al., 2020). Thus, future studies should survey adolescents from different cultural groups and examine whether similar findings can be obtained in countries with an individualistic culture. Lastly, future studies should expand the scope of factors that may serve as mediators or moderators. According to the theoretical model of the “stress – sleep” relationship, emotional response is just one form of stress responses (Yan et al., 2010). Thus, future studies should examine whether other forms of stress responses (e.g., cognitive response, behavioral response) can significantly mediate the relationship between parental phubbing and adolescent sleep quality problems.

## 7. Conclusion

In summary, parental phubbing is an important factor that influences adolescent sleep quality problems. Negative emotions mediate the relationship between parental phubbing and adolescent sleep quality problems. And self-control moderated the effect of parental phubbing on adolescent negative emotions. Specifically, the mediating effect of negative emotions was more significant for adolescents low in self-control relative to those high in self-control. Therefore, in order to help adolescents decrease sleep quality problems, we can reduce their parental phubbing, reduce their negative emotions, and maintain their moderate self-control.

## Data availability statement

The raw data supporting the conclusions of this article will be made available by the authors, without undue reservation.

## Ethics statement

The studies involving human participants were reviewed and approved by Research Ethics Committee of School of Educational Science, Xinyang Normal University. Written informed consent to participate in this study was provided by the participants’ legal guardian/next of kin.

## Author contributions

QD and YZ designed the work and were responsible for the overall development of this study, including the planning of sample collection, data analysis, writing, and polishing of the manuscript. QD, SD, and YZ were responsible for revising the manuscript and made a great contribution to the final acceptance of the manuscript. QD provided the manuscript fee. All authors contributed to the article and approved the submitted version.

## Funding

This work was supported by the Youth Project of the National Social Science Foundation of China (20CSH098).

## Conflict of interest

The authors declare that the research was conducted in the absence of any commercial or financial relationships that could be construed as a potential conflict of interest.

## Publisher’s note

All claims expressed in this article are solely those of the authors and do not necessarily represent those of their affiliated organizations, or those of the publisher, the editors and the reviewers. Any product that may be evaluated in this article, or claim that may be made by its manufacturer, is not guaranteed or endorsed by the publisher.
